# Loss of Splicing Factor SRSF3 Impairs Lipophagy Through Ubiquitination and Degradation of Syntaxin17 in Hepatocytes

**DOI:** 10.1016/j.jlr.2023.100342

**Published:** 2023-02-08

**Authors:** Yun Li, Tao Wang, Qiumin Liao, Xiaoting Luo, Xing Wang, Shu Zeng, Mengyue You, Yaxi Chen, Xiong Z. Ruan

**Affiliations:** 1 Centre for Lipid Research & Key Laboratory of Molecular Biology for Infectious Diseases (Ministry of Education), the Second Affiliated Hospital, Chongqing Medical University, Chongqing, China; 2 John Moorhead Research Laboratory, Centre for Nephrology, University College London Medical School, Royal Free Campus, University College London, London, United Kingdom

**Keywords:** lipid droplets, liver, triglyceride, obesity, lipid/oxidation, NAFLD, autophagic flux, proteasome, SNARE protein, E3 ligase, ATP, adenosine triphosphate, HFD, high-fat diet, NAFLD, nonalcoholic fatty liver disease, ORO, Oil red O, PA, palmitic acid, SIAH1, seven in absentia homolog 1, SRSF3, serine/arginine-rich splicing factor 3, STX17, syntaxin 17, TG, triglyceride

## Abstract

Lipid accumulation in hepatocytes is the distinctive characteristic of nonalcoholic fatty liver disease. Serine/arginine-rich splicing factor 3 (SRSF3) is highly expressed in the liver and expression decreases in high-fat conditions. However, the role of SRSF3 in hepatic lipid metabolism needs to be clarified. Here, we showed that loss of SRSF3 was associated with lipid accumulation. We determined that SRSF3 regulated lipophagy, the process of selective degradation of lipid droplets by autophagy. Mechanistically, loss of SRSF3 impaired the fusion of the autophagosome and lysosome by promoting the proteasomal degradation of syntaxin 17 (STX17), a key autophagosomal SNARE protein. We found that ubiquitination of STX17 was increased and upregulation of seven in absentia homolog 1 was responsible for the increased posttranslational modification of STX17. Taken together, our data primarily demonstrate that loss of SRSF3 weakens the clearance of fatty acids by impairing lipophagy in the progression of nonalcoholic fatty liver disease, indicating a novel potential therapeutic target for fatty liver disease treatment.

## Graphical abstract

**Figure:**
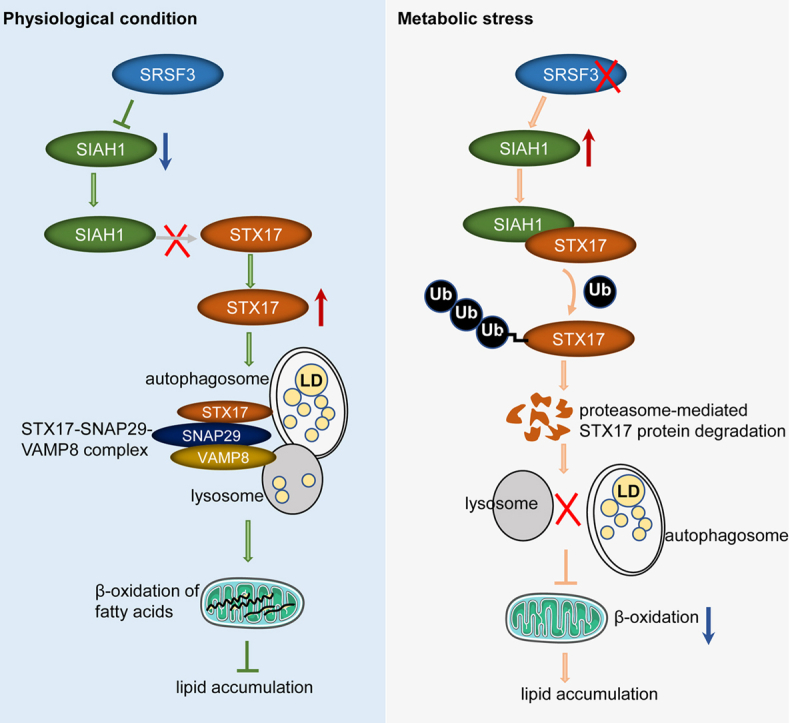

Nonalcoholic fatty liver disease (NAFLD) is the most prevalent chronic liver disease, which is characterized by extremely complex pathogenetic mechanisms and multifactorial etiology (1, 2). NAFLD encompasses a broad spectrum beginning with steatosis and is frequently associated with T2DM, obesity, and dyslipidemia (3). The long-term sustainability of diet and exercise in NAFLD treatment is poor (4). There are no licensed treatments for NAFLD (5, 6). Elucidating the molecular mechanisms underlying the steatosis in the progression of NAFLD will be beneficial to look for therapeutic targets for fatty liver diseases.

Serine/arginine-rich splicing factor 3 (SRSF3) is the smallest member of the serine/arginine-rich family proteins that function to regulate alternative splicing of pre-mRNA (7, 8), export of mature mRNA from the nucleus (9, 10), stability of mRNA (11) and translational control (12, 13, 14). SRSF3 is abundant in the liver. With the progression of metabolic liver disease, SRSF3 was decreased gradually (15, 16). Previous studies have pointed out that SRSF3 regulates the alternative splicing of the insulin receptor gene and is involved in glucose metabolism (17). However, the role of SRSF3 in hepatic lipid metabolism needs to be clarified.

Lipophagy is identified to play a central role in the breakdown of hepatic lipid droplet-stored triglyceride (TG) (18, 19, 20). Our team previously elucidated that lipophagy is regulated by fatty acid translocase (CD36) in hepatocytes and prevents the progression of NAFLD (21). Emerging evidence suggests that impaired lipophagy is involved in hepatic lipid metabolism (22, 23, 24, 25). Decreased autophagosome-lysosome fusion blocks the degradation of lipid droplets engulfed by autophagosome. Syntaxin 17 (STX17) is the key autophagosomal SNARE protein that mediates the fusion of autophagosome and lysosome by forming STX17-SNAP29-VAMP8 SNARE complex. The present study demonstrates a new function of SRSF3 that regulates lipophagy in hepatocytes and explores the underlying mechanisms both in vitro and in vivo. SRSF3 loss significantly increases the ubiquitination and degradation of STX17, blocks the fusion of autophagosome and lysosome and impairs lipophagy. The ubiquitination modification of STX17 is attributed to an increase of E3 ubiquitin ligase seven in absentia homolog 1 (SIAH1) at the condition of SRSF3 loss. The reduction of lipophagy weakens the clearance of fatty acids. It aggravates lipid accumulation in the liver, in addition to regulating the alternative splicing of genes related to metabolism in NAFLD. These results suggest that targeting SRSF3 may be a potential therapeutic strategy for NAFLD.

## Materials and methods

### Experimental animals

Eight-week-old male C57BL/6J mice were fed with a normal chow diet (Research Diets, D12450B) or high-fat diet (HFD) (Research Diets, D12492) for 14 weeks. All mice were housed in a temperature-controlled environment and 12 h: 12 h light: dark cycle with free access to diet and water. Before sacrifice, mice with free access to water were deprived of food overnight. Animal care and experimental procedures were performed with approval from the animal care committees of Chongqing Medical University.

### Cell culture and transfection

Human hepatic cell lines HepG2 and Huh7, human immortalized hepatocyte THLE-3 cells, and mouse hepatocyte AML-12 cells were selected. Cells were kept in DMEM (Gibco) supplemented with 10% FBS (NSERA). Cells were maintained in a 37°C incubator with 5% CO_2_ and grown to 70–90% confluence and were collected for analysis unless otherwise noted. Primary hepatocytes were isolated from livers of C57BL/6J mice aged 8–10 weeks using a modified two-step collagenase perfusion method. Briefly, buffer A (2.5 mM EGTA, 0.1% glucose, and 2% penicillin/streptomycin) and buffer B (Liver Digest Medium, Gibco, 17703034) were injected through the hepatic portal vein. Then, the isolated mouse hepatocytes were plated in 6-well plates for analysis. All analyses in cells were performed after treatment with 0.2 mM palmitic acid (PA) (Sigma-Aldrich, P9767) for 24 h. siRNAs were produced by OBIO Technology (Shanghai, China). The sequences of siRNA targeting human *SRSF3* were as follows: ugaugcaguccgagagcuatt (5′–3′) and uagcucucggacugcaucatt (5′–3′). The sequences of siRNA targeting human *SIAH1* were as follows: atgagccgucagacugcuaca (5′–3′) and uguag-cagucugacggcucau (5′–3′). The sequences of siRNA-targeting mouse *Srsf3* were as follows: gguuacaaauuguuguuuatt (5′–3′) and uaaacaacaauuuguaacctt (5′–3′). siRNA was transfected using Lipofectamine RNAiMAX (Invitrogen, 92008) according to the manufacturer’s instruction. For plasmid transfection, cells were transfected with the desired plasmid using Lipo3000 and P3000 (Invitrogen, 92008) in Opti-MEM (Gibco, 2193200) according to the manufacturer’s instruction. After 12–18 h of incubation, the culture medium was changed to a complete culture medium, and the experiment was conducted after 48 h.

### Immunofluorescence

Cells were seeded on coverslips prior to treatment. Liver-frozen sections or cells were washed with PBS, fixed with 4% paraformaldehyde for 25 min. Cells were infiltrated with 100% methanol for 10 min at a temperature of minus 20°C and blocked for 1 h. Cells were incubated with primary antibody overnight at 4°C and incubated with Alexa Fluor 568 (Thermo Fisher Scientific, A11036) for 2 h at room temperature. Cells were stained with DAPI (Thermo Fisher Scientific, D1306) for 5 min. For lipid staining, cells were stained with Bodipy 493/503 (Invitrogen) for 30 min at room temperature. Lysosome was tracked using LysoTracker (Life Technology, L7528). Coverslips were visualized by using a Leica confocal microscope.

### Measurements of mitochondrial respiration

Oxygen consumption was measured on a Seahorse XF24 analyser at 37°C. Seahorse XFe24 FluxPak mini (102342-100) and Seahorse XF base medium (102353-100) was purchased from Agilent (California). Oligomycin, carbonyl cyanide 4-(trifluoromethoxy) phenylhydrazone (FCCP), and Rotenone/Antimycin-A were purchased from Sigma. Forty-eight hours before the assay, 40,000 siSRSF3 or siNeg HepG2 cells were seeded in a Seahorse XF24 analyser plate. After inducing enough lipid droplets by pretreating HepG2 cells with 0.2 mM PA for 24 h, we replaced the medium with Seahorse XF base medium and measured the mitochondrial respiration in the basal state and after 1 μM oligomycin, 2 μM FCCP, and 0.5 μM Rotenone/Antimycin-A treatment. Further, in another assay, instead of pretreating cells with PA to produce lipid droplets, we added excessive exogenous PA and then measured oxygen consumption. Adenosine triphosphate (ATP) production, basal respiration, maximal respiration, and spare respiratory capacity were calculated, respectively. Cells were lysed and protein concentration was taken for normalization.

### GFP-RFP-LC3 assay

Cells were transduced with mRFP-GFP-LC3 adenoviral vectors (HanBio Technology, Shanghai, China) and grown for 2 h. The principle of the assay is based on the different pH stability of red and GFPs. The mRFP signal did not change significantly in acidic conditions, whereas the GFP signal could be quenched under the acidic condition (pH < 5) inside the lysosome. In red- and green-merged images, autophagosomes are shown as yellow puncta, while autolysosomes are shown as red puncta. LC3 puncta were examined with a Leica confocal microscope.

### Histological analysis

Liver tissue was fixed in 4% paraformaldehyde in PBS. The tissue section was stained by H&E and Oil red O (ORO).

### Polysome analysis

Polysomal analysis was performed with reference to previous literature (26, 27). Cells were harvested and polysomes were extracted with 0.3 ml low-salt buffer (20 mM Tris-HCl, pH 7.4, containing 10 mM NaCl, and 3 mM MgCl2) prior to homogenization in 0.1 ml low-salt buffer containing 1.2% (v/v) Triton X-100 and 0.2 M sucrose. The lysate was applied to a 5–45% linear sucrose gradient and ultracentrifuged at 25 0000×g for 2 h at 4°C. The absorbance at 254 nm was measured by using a UV spectrophotometer. *STX17* and *GAPDH* were detected by RT-PCR and agarose gel electrophoresis. Specific primer sequences were as follows: *STX17* forward: tggtggagcatttcatactactg; *STX17* reverse: ttggttccctcttcaacattc; *GAPDH* forward: atcactgccacccagaagact; *GAPDH* reverse: ctgttgaagtcagaggagaccac.

### Quantitative PCR

Total RNA was extracted using TRIzol reagent. Then, 1μg total RNA was reverse-transcribed with PrimeScript RT Reagent Kit (TaKaRa, Japan). Quantitative PCR (qPCR) was performed using SYBR Green PCR Mix kit (TaKaRa). Specific primer sequences used for qPCR were listed in supplementary Table S1. β-actin was used for normalization. Fold change was calculated using 2-ΔΔCt.

### Immunoblotting

Cells and mouse liver tissues were lysed with RIPA lysis buffer. Whole-cell extracts containing 20 μg protein per lane were dissolved on an acrylamide gel and blotted wetly onto the PVDF membrane. Membranes were blocked for 30 min at room temperature and incubated with specific antibodies overnight at 4°C. The primary antibodies were listed in supplementary Table S2. Detection was achieved by using an HRP-conjugated secondary antibody in conjunction with the ECL reagent. Afterwards, membranes were exposed and quantification of signals was quantified using Image J (https://imagej.net/ij/index.html).

### Protein stability

To measure protein stability, cells were treated with 500 μg/ml cycloheximide (Selleck, S7418) for the indicated time periods. STX17 protein was measured by Western blotting.

### Immunoprecipitation

For immunoprecipitation, equal amounts of lysate proteins were incubated with the antibodies overnight at 4°C. Protein A/G Mix Magnetic Bead (Millipore) were added and incubated for 2 h. After washing with cold lysis buffer, the bound proteins were eluted by boiling for 5 min in the SDS sample buffer. The supernatants were analyzed by SDS-PAGE and immunoblotted with the respective antibody. For ubiquitin detection, MG132 (MedChemExpress, HY-13259) was used to inhibit protein degradation.

### Statistical analysis

All cell experiment data represented at least three independent experiments and were shown as individual data points and mean ± SD. The difference between two groups was statistically analyzed using Student’s *t*-test in GraphPad Prism version 8 (https://www.graphpad.com/). The statistical significance was given in the figure legend.

## Results

### SRSF3 expression is decreased under high-fat condition and loss of SRSF3 was associated with lipid accumulation

**Fig. 1:**
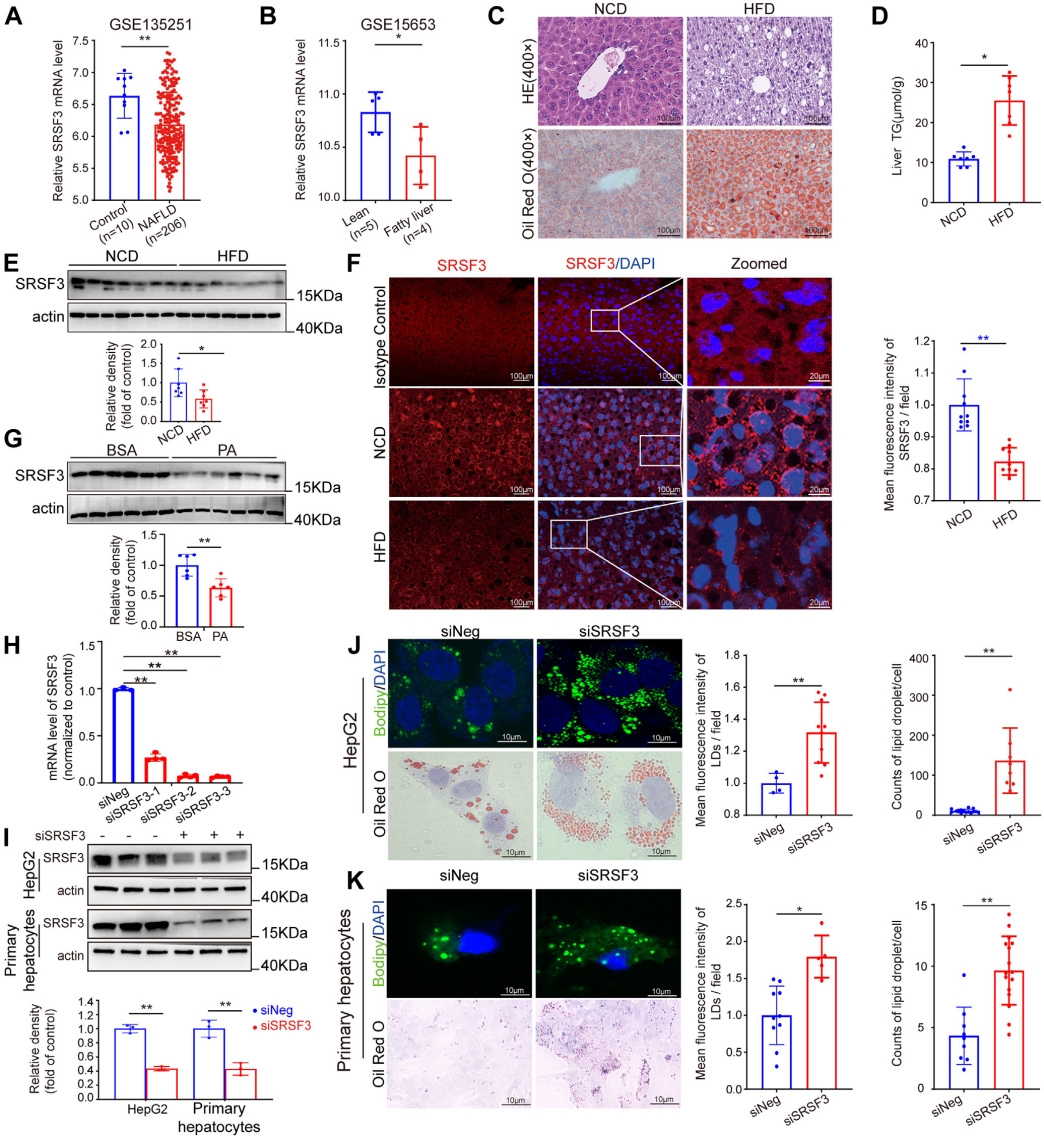
SRSF3 is decreased under high-fat condition and SRSF3 knockdown promotes lipid accumulation in hepatocytes. SRSF3 expression in liver samples of patients with NAFLD and control individuals (A) and SRSF3 in fatty liver and control (B). Mice were fed with NCD or HFD for 14 weeks (n = 7). C: H&E (upper panel, ×400) and Oil Red O staining (lower panel, ×400) of liver sections. D: Triglyceride in liver tissues. E: Analysis of SRSF3 in mouse livers by Western blotting and immunofluorescent staining (F). G: HepG2 cells were treated with 0.2% BSA or 0.2 mM PA and SRSF3 was analysed by Western blotting (n = 6). SRSF3 was knocked down with siRNA and measured by quantitative PCR (qPCR) (n ≥ 3) (H) and Western blotting (n = 3) (I). Lipid staining with Bodipy 493/503 (upper panel) and Oil Red O (lower panel) after knocking-down SRSF3 in HepG2 cells (J) or in isolated mouse primary hepatocytes (K) under 0.2 mM PA treatment. Data are shown as individual data points and mean ± SD and ∗*P* < 0.05, ∗∗*P* < 0.01. NAFLD, nonalcoholic fatty liver disease; SRSF3, Serine/arginine-rich splicing factor 3; PA, palmitic acid.

Data about SRSF3 in the fatty liver were retrieved from the Gene Expression Omnibus database (http://www.ncbi.nlm.nih.gov/geo/, GSE135251, 15653) (28, 29). We found that SRSF3 were significantly reduced in biopsies of human fatty liver disease (Fig. 1A, B). We placed eight-week-old C57BL/6 mice on a HFD (60% fat) for 14 weeks to establish a NAFLD model. The mice on HFD were gaining weight faster and more (supplemental Fig. S1A, B). Morphologically, H&E and ORO staining of liver slices demonstrated that evident steatosis existed in the livers of HFD-treated mice (Fig. 1C). TG content was significantly higher in HFD-treated mouse livers than that in normal chow diet-treated mouse livers (Fig. 1D). To determine SRSF3 expression in mice livers of NAFLD and corresponding control, we performed Western blotting and immunofluorescent staining. We found that SRSF3 expression was decreased in the liver of NAFLD mice (Fig. 1E, F). Further, we also observed a significant decrease of SRSF3 protein in HepG2 cells with 0.2 mM PA treatment for 24 h (Fig. 1G). This suggested that SRSF3 may be negatively correlated with lipid accumulation in the liver. *SRSF3* was knocked down in vitro with three pairs of siRNAs-targeting *SRSF3* (Fig. 1H). The efficiency of knockdown was verified in HepG2 and isolated mouse primary hepatocytes (Fig. 1I). Lipid was accumulated significantly after knocking-down SRSF3 in HepG2 and mouse primary hepatocytes by staining with Bodipy 493/503 and ORO (Fig. 1J, K). This suggested that loss of SRSF3 was associated with lipid deposition in hepatocytes.

### Loss of SRSF3 impairs autophagic flux in hepatocytes

**Fig. 2:**
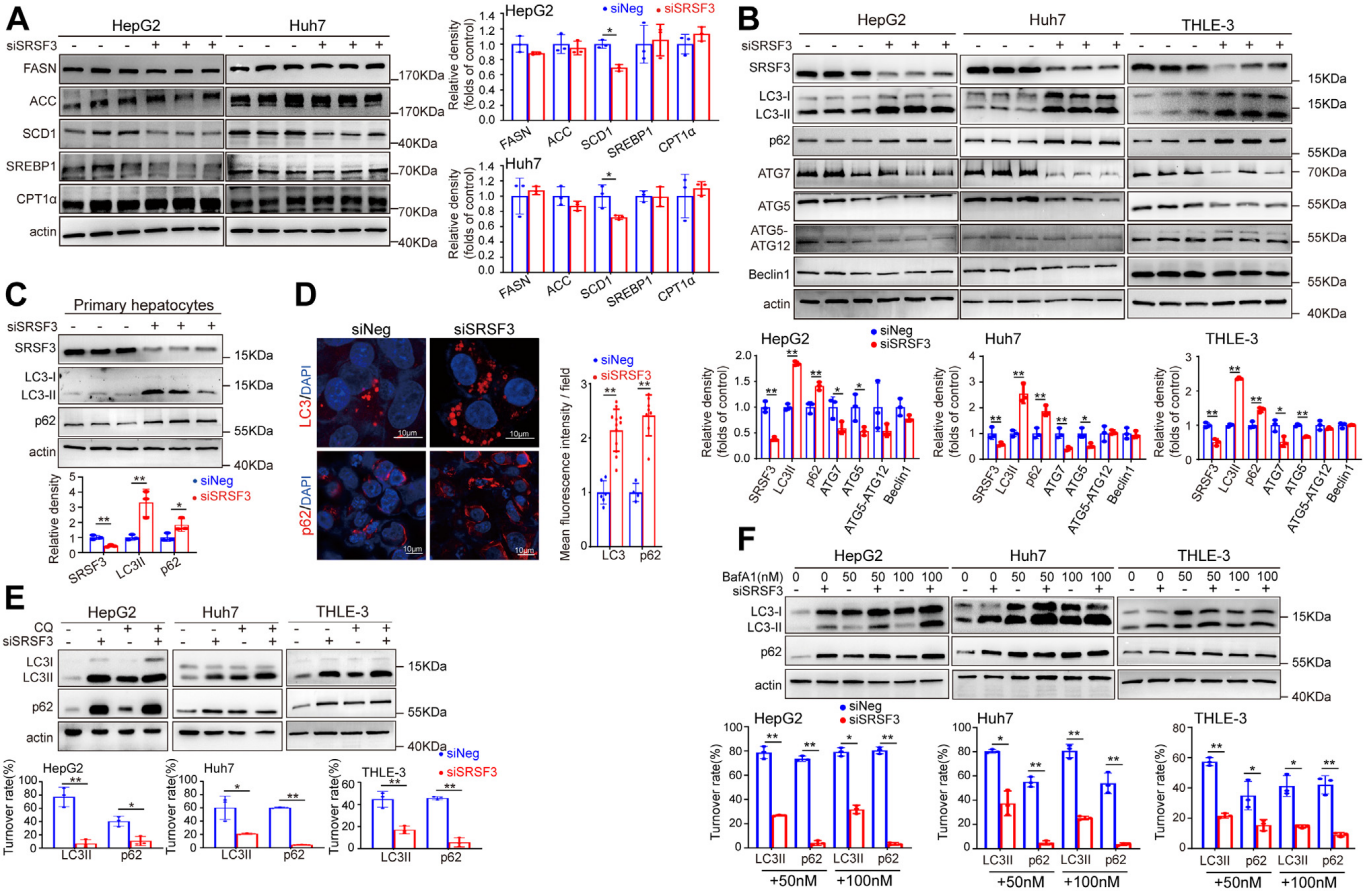
SRSF3 knockdown impairs autophagic flux in hepatocytes. SRSF3 was knocked down in HepG2, Huh7, THLE-3 cell lines and isolated mouse primary hepatocytes (n = 3). A: Analysis of lipogenic protein and CPT1A. B, Analysis of p62/SQSTM1 and ATG proteins in HepG2, Huh7, THLE-3 cell lines by Western blotting. C: p62/SQSTM1 and LC3 in primary hepatocytes. D: p62 and LC3 was analyzed by immunofluorescent staining and mean fluorescence intensity per field was calculated. p62 and LC3 was detected after treatment with 10μM chloroquine (CQ) (E) and 50 nM or 100 nM bafilomycin A1 (Baf A1) (F) by immunoblotting (n = 3). Data are shown as individual data points and mean ± SD and ∗*P* < 0.05, ∗∗*P* < 0.01. SRSF3, Serine/arginine-rich splicing factor 3.

To explore the underlying mechanism of SRSF3-regulating lipid accumulation, we detected proteins involved in de novo lipogenesis and β-oxidation. The data showed that the transcription factor SREBP1 and its downstream lipogenic enzymes FASN, ACC, and SCD1 were not significantly increased under the condition of SRSF3 deletion. The fatty acid shuttling enzyme CPT1A was not decreased (Fig. 2A). This suggested that the lipid accumulation induced by SRSF3 knockdown was not due to de novo lipogenesis or β-oxidation. Autophagy of lipid droplets, a process known as lipophagy, is an important process of hepatic lipid catabolism (18, 30, 31). To evaluate the role of SRSF3 in autophagy under high-fat conditions, we detected several autophagy-related (ATG) proteins and p62/SQSTM1. Since adapter protein p62/SQSTM1 accumulates when autophagy is inhibited, and decreased levels can be observed when autophagy is induced, p62 may be used as an autophagy degradation marker to study autophagic flux (32). We observed an obvious accumulation of p62 and LC3Ⅱ in HepG2, Huh7, THLE-3 cell lines, and isolated mouse primary hepatocytes (Fig. 2B, C), suggesting that the autophagy degradation may be inhibited. We further confirmed this result by immunofluorescence staining (Fig. 2D). In order to clarify whether the increased p62 and LC3Ⅱ were due to an increase of production or the accumulation caused by a decreased degradation at the late stage of autophagic flux, we treated HepG2, Huh7, THLE-3 cells with autophagy inhibitor chloroquine or bafilomycin A1 after knocking-down SRSF3. We found that in the condition of SRSF3 deletion, p62 protein was not increased with the treatment of the two autophagy inhibitors above, suggesting that p62 flux had been blocked by SRSF3 knockdown and loss of SRSF3 was sufficient to induce a blockage of degradation (Fig. 2E, F). Similarly, LC3Ⅱ was also accumulated (Fig. 2E, F). Unexpectedly, under the SRSF3 knockdown condition in Huh7 and HepG2 cell lines, LC3Ⅱ was still increased slightly with bafilomycin A1 treatment, suggesting that production may also be also involved in the accumulation of LC3Ⅱ. We found that LC3 mRNA was induced by SRSF3 knockdown (supplemental Fig. S1C). The increase of LC3 transcription may be partly involved in the accumulation of LC3. However, ATG5 and ATG7, two critical proteins to the conversion of LC3-Ⅰ to LC3-Ⅱ, were decreased. We speculated that the conversion of LC3-Ⅰ to LC3-Ⅱ was inhibited and the increase of LC3-II content was mainly due to the accumulation caused by the decreased degradation of autophagy. Generally, the degradation system of autophagy was indeed impaired and late autophagic flux was blocked in the condition of SRSF3 loss in hepatocytes.

### Loss of SRSF3 reduces lipophagy followed by severely attenuated β-oxidation

**Fig. 3:**
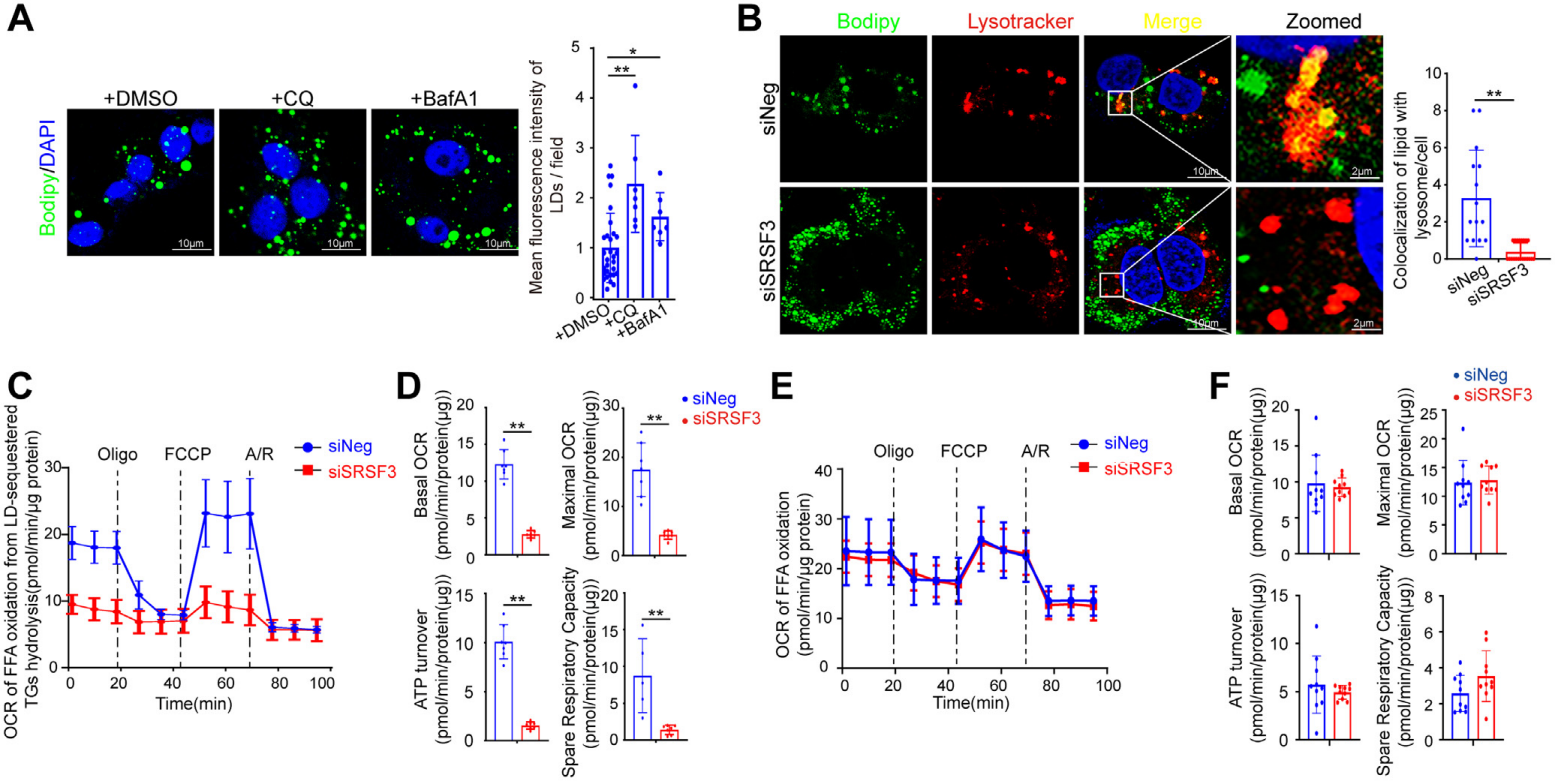
SRSF3 knockdown attenuates lipophagy and β-oxidation. A: Lipid staining with Bodipy 493/503 after treatment with CQ and Baf A1 and mean fluorescence intensity per field was calculated. B: Colocalization of lipid with lysosome (Lysotracker/Bodipy) in HepG2 cells under the condition of 0.2 mM PA treatment. At least fifteen cells were used for statistical analysis of the number of colocalization puncta per cell. C: After inducing enough lipid droplets by pretreating cells with 0.2 mM PA for 24 h, mitochondrial respiration was measured. D: Basal respiration, ATP production, maximal respiration, and spare respiratory capacity were calculated according to OCR in (C) (n ≥ 5). E: Instead of pretreating cells with PA for 24 h to produce lipid droplets, excessive exogenous PA was added and mitochondrial respiration was measured. F: Basal respiration, ATP production, maximal respiration, and spare respiratory capacity were calculated according to OCR in (E) (n = 10). Data are shown as individual data points and mean ± SD and ∗*P* < 0.05, ∗∗*P* < 0.01. SRSF3, Serine/arginine-rich splicing factor 3; OCR, oxygen consumption rate; ATP, adenosine triphosphate; PA, palmitic acid.

The autophagolysosomal system engulfs and degrades lipid droplets. Lipid was accumulated significantly with the treatment of lysosomal inhibitors chloroquine or bafilomycin A1 (Fig. 3A), suggesting lysosome-based degradation was crucial to lipid homeostasis. So, we further observed the lysosomal lipid in siSRSF3 and controlled HepG2 cells after loading 0.2 mM PA for 24 h (Fig. 3B). The data showed that the colocalization of lipid with lysosome (Bodipy/Lysotracker) was decreased after knocking-down SRSF3, suggesting that lipophagy was impaired by SRSF3 knockdown. Mitochondria play pivotal roles in cellular energy metabolism (33). After inducing enough lipid droplets by pretreating HepG2 cells with 0.2 mM PA for 24 h, we changed the medium and measured the mitochondrial respiration. The data showed that the oxygen consumption rate was decreased after knocking-down SRSF3 in HepG2 cells (Fig. 3C). Basal respiration, ATP production, maximal respiration and spare respiratory capacity were also reduced by SRSF3 knockdown (Fig. 3D). Further, instead of pretreating cells with 0.2 mM PA for 24 h to produce lipid droplets, we added excessive exogenous palmitic acid, and then measured oxygen consumption. We found that there was no significant difference of oxygen consumption rate between the knockdown cells and the control cells, as well as basal respiration, ATP production, maximal respiration, and spare respiratory capacity (Fig. 3E, F). This suggested that loss of SRSF3 did not impair mitochondrial oxidative phosphorylation. Therefore, the impaired oxidative metabolism induced by SRSF3 loss was attributed to the fact that lipid droplets-sequestered TGs hydrolysis by lipophagy was blocked and following by a reduction of FFA content.

### Loss of SRSF3 blocks the fusion of autophagosome and lysosome via reducing STX17 protein

**Fig. 4:**
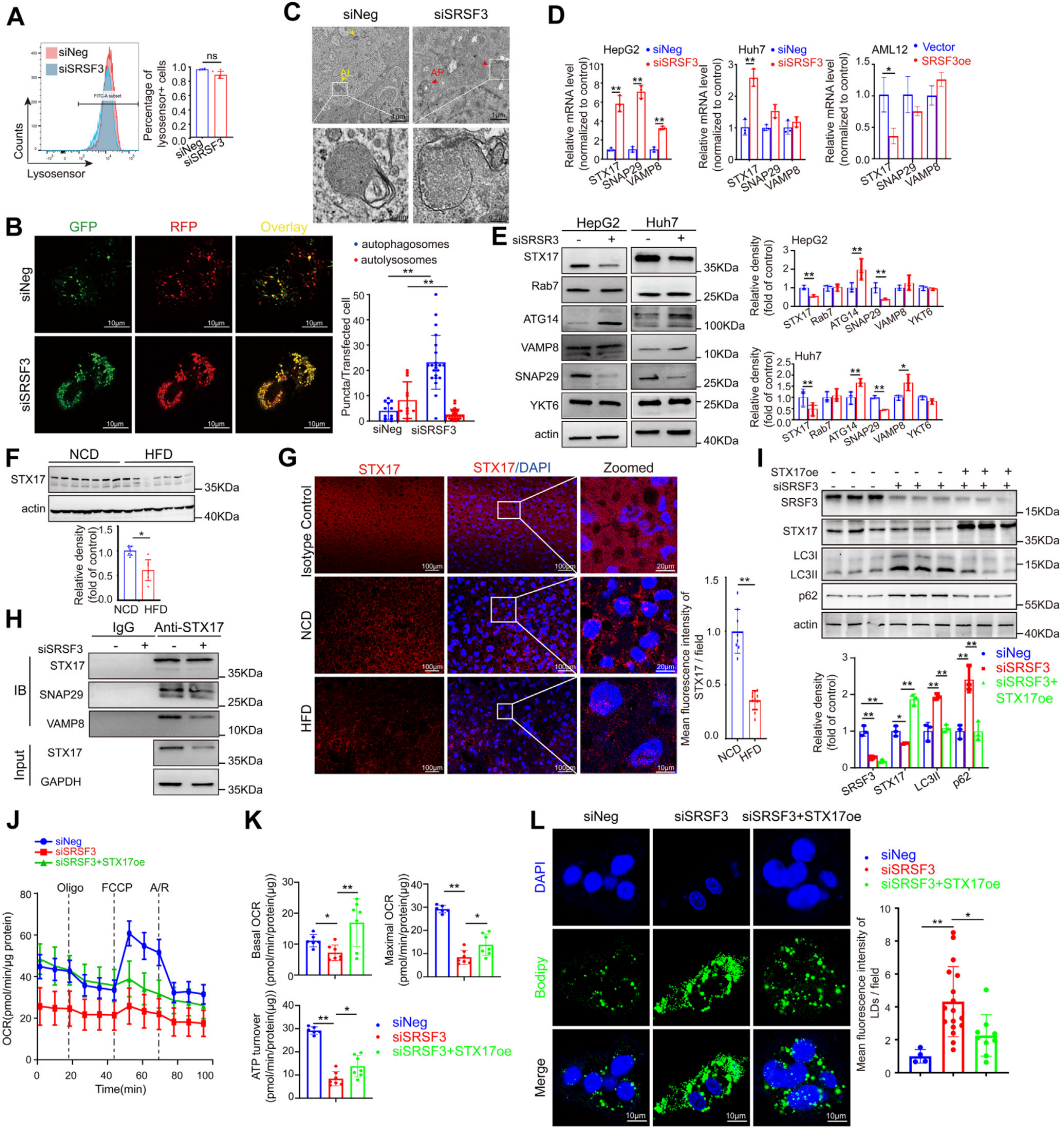
Loss of SRSF3 blocks the fusion of autophagosome and lysosome via reducing STX17 protein. A: Detection of lysosome pH by flow cytometry. B: Analysis of double fluorescent mRFP-EGFP-LC3 fusion protein expression to detect autophagic flux (Yellow puncta represent autophagosomes with both red and green fluorescence. Red puncta represent autolysosomes with only red fluorescence). At least twelve cells were used for statistical analysis of the number of puncta per cell. C: Electron microscopy of control or SRSF3 knockdown HepG2 cells. Large autophagolysosomes form in control cells and accumulated autophagosomes in SRSF3 knockdown HepG2 cells. AL, autophagolysosome; AP, autophagosome. mRNA (D) and protein (E) detection of fusion-related genes by qPCR and Western blotting, respectively. Analysis of SRSF3 in mouse livers by Western blotting (n = 7) (F) and immunofluorescent staining (G). H: Analysis of STX17-SNAP29-VAMP8 complex by coimmunoprecipitation. STX17 was overexpressed. p62 and LC3 were analysed (I), mitochondrial respiration was measured (J), (K) basal respiration, ATP production and maximal respiration were calculated according to OCR in (J) (n ≥ 6), and lipid was stained with Bodipy 493/503 (L). Data are shown as individual data points and mean ± SD and ∗*P* < 0.05, ∗∗*P* < 0.01. SRSF3, Serine/arginine-rich splicing factor 3.

Disruption of lysosomal function or a fusion failure of autophagosome and lysosome impairs autophagic flux and reduces the degradation of autophagic cargoes. Transcription factor EB plays an important role in the transcription of lysosome-related genes. Nuclear transcription factor EB protein and mRNA expressions of *LAMP1*, *cathepsin B (CTSB)* and *cathepsin D (CTSD)* were induced by SRSF3 knockdown (supplemental Fig. S1D, E). Unexpectedly, the protein levels of CTSD and CTSB were decreased, suggesting that the mRNA and protein expressions of the genes above were diverging (supplemental Fig. S1F). We next found that loss of SRSF3 did not affect the lysosome pH detected by flow cytometry (Fig. 4A). mRFP-GFP-LC3 adenovirus reporter is used for autophagy flux analysis. mRFP-GFP-LC3 adenovirus reporter fluoresces both red and green (shown as yellow) in autophagosomes and only red in autolysosomes due to the quenching of GFP in acidic conditions. In the SRSF3 knockdown cells, we observed an increased number of yellow puncta and a smaller number of red puncta, suggesting that the fluorescence of GFP had not been quenched (Fig. 4B). We speculated that a fusion failure occurred. Ultrastructural analysis by electron microscopy confirmed this speculation: large autophagolysosomes form existed in the control cells, and instead, accumulated autophagosomes appeared in the SRSF3 knockdown cells (Fig. 4C). These experiments demonstrated that SRSF3 loss blocked the fusion of lysosomes and autophagosomes and thereby impaired autophagic flux. STX17-SNAP29-VAMP8 SNARE complex is crucial to the fusion of autophagosome and lysosome (34). We detected the mRNA and protein expressions of STX17, SNAP29, and VAMP8. The data showed that SRSF3 negatively regulated *STX17* mRNA expression (Fig. 4D). However, the key autophagosomal SNARE protein STX17 was significantly decreased after knocking-down SRSF3 (Fig. 4E). In vivo, STX17 was also decreased in the livers of HFD-treated mice by Western blotting and immunofluorescent staining (Fig. 4F, G). In addition, we demonstrated that the STX17-SNAP29-VAMP8 SNARE complex was decreased in the SRSF3 knockdown cells by immunoprecipitation (Fig. 4H). We constructed plasmids overexpressing STX17. The accumulations of p62 and LC3Ⅱ were relieved significantly (Fig. 4I). The decreased mitochondrial respiration was rescued and the lipid accumulation was also alleviated after overexpressing STX17 (Fig. 4J, K and L). Therefore, the reduction of STX17 was the key to mediating the blockage of fusion in the SRSF3 deficiency condition.

### Loss of SRSF3 promotes the ubiquitination and degradation of STX17 through upregulating the E3 ligase SIAH1

**Fig. 5:**
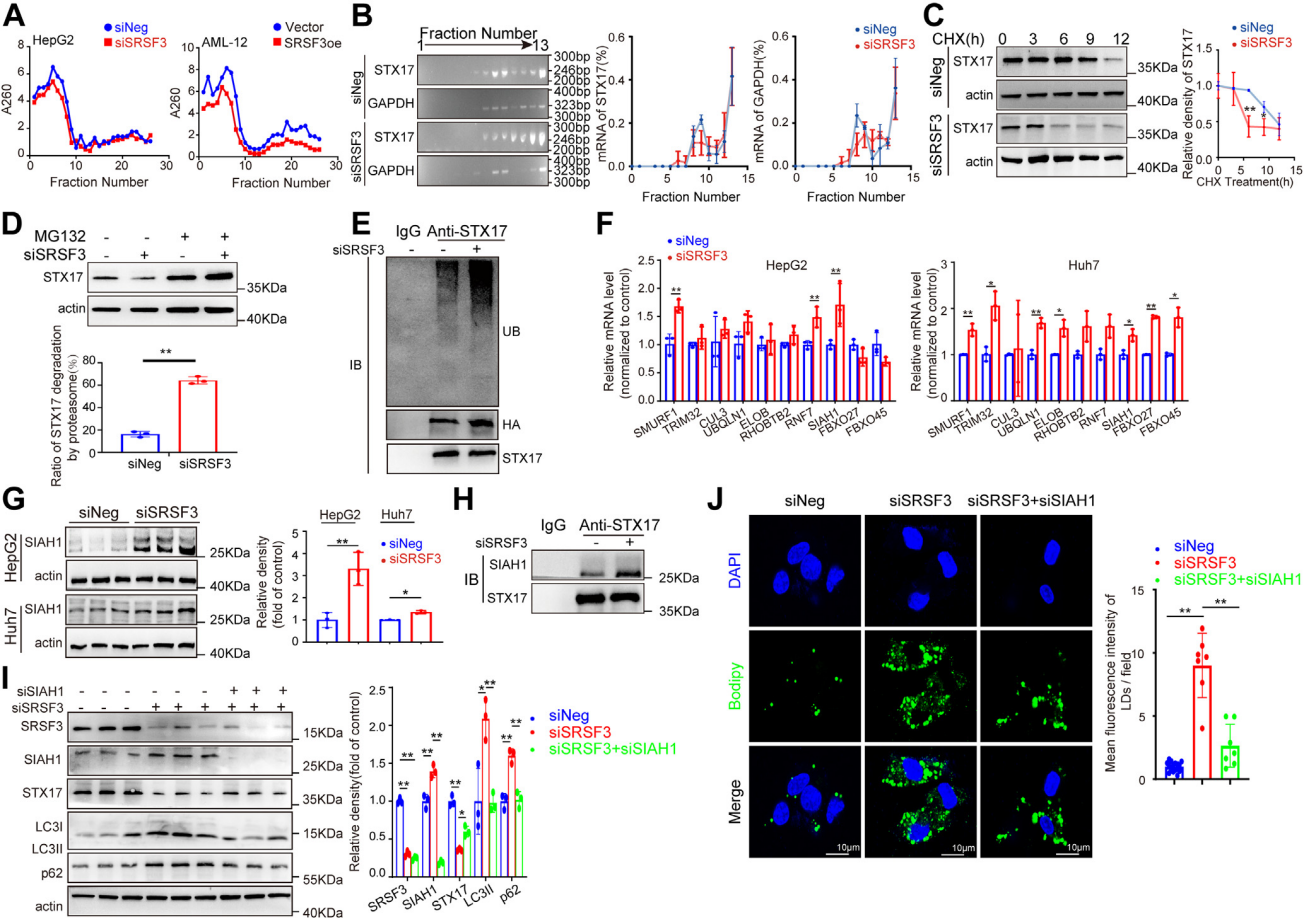
Loss of SRSF3 promotes the ubiquitination and degradation of STX17 through upregulating the E3 Ligase SIAH1. A: Sucrose gradient polysome fractionation was used to extract ribosomal fractions. B: The relative association of *STX17* mRNA with different ribosomal fractions was detected by RT-PCR and agarose gel electrophoresis. Analysis of STX17 after treatment with 500μg/ml cycloheximide (CHX) for the indicated time (n = 3) (C) or with 40 μM MG-132 for 6 h (n = 3) (D). E: The ubiquitination detection of STX17 by immunoprecipitation. F: mRNA levels of several E3 ubiquitin ligase genes (n = 3). G: SIAH1 protein analysis (n = 3). H: Interaction of STX17 and SIAH1 was analysed by coimmunoprecipitation. SIAH1 was knocked down with siRNA. STX17, p62, and LC3 were detected by Western blotting (n = 3) (I) and lipid was stained with Bodipy 493/503 (J). Data are shown as individual data points and mean ± SD and ∗*P* < 0.05, ∗∗*P* < 0.01. SRSF3, Serine/arginine-rich splicing factor 3; STX17, Syntaxin 17; SIAH1, seven in absentia homolog 1.

Diverging mRNA and protein expression of STX17 in hepatocytes suggested that decreased protein stability or increased translational repression may be the reason for the decrease of STX17 protein. To clarify whether SRSF3 modulates the translational efficiency of STX17, we used sucrose gradient polysome fractionation to measure the relative association of *STX17* mRNA with different ribosomal fractions. Several fractions were collected and identified, and the data showed that gain or loss of SRSF3 did not affect the efficiency of RNA translation in hepatocytes (Fig. 5A). The *STX17* mRNA in each fraction was measured by RT-PCR and agarose gel electrophoresis. The data showed that the translation of *STX17* was not inhibited by SRSF3 knockdown (Fig. 5B). The cycloheximide chase assay showed that the half-life of STX17 protein was shortened significantly by SRSF3 knockdown (Fig. 5C). Subsequently, we used MG-132 (a selective 26S proteasomal inhibitor) to address the proteasomal degradation of STX17. The ratio of STX17 degradation by the proteasome in SRSF3 knockdown cells was significantly higher than that in control cells (Fig. 5D). The ubiquitination of STX17 was increased in SRSF3 knockdown cells when STX17 was immunoprecipitated using a specific antibody (Fig. 5E). To identify the E3 ubiquitin ligase responsible for STX17 ubiquitination, we screened several genes by qPCR. Of note, the mRNA level of *SIAH1*, a canonical RING finger ubiquitin ligase, was significantly increased in both SRSF3 knockdown HepG2 and Huh7 cells (Fig. 5F). SIAH1 protein was also induced by SRSF3 knockdown (Fig. 5G). Further, the direct binding of SIAH1 and STX17 was promoted markedly in the condition of SRSF3 deficiency by the coimmunoprecipitation assay (Fig. 5H). We transfected siRNA-targeting SIAH1 and found that the degradation of STX17 was successfully rescued and the accumulations of p62 and LC3Ⅱ were relieved significantly (Fig. 5I). The lipid accumulation was also alleviated after downregulating SIAH1 (Fig. 5J).

## Discussion

RNA-binding protein SRSF3 has been ascribed to a number of cellular functions, including as a proto-oncogene or a potential suppressor in many types of cancer. SRSF3 acts as an alternative splicing factor in hepatocyte differentiation (15, 17, 35). SRSF3 is highly expressed in the liver and is decreased with high-fat treatment. However, the underlying mechanism of SRSF3-regulating lipid metabolism is not well understood. In this study, we first demonstrated that impaired lipophagy induced by loss of SRSF3 played an important role in lipid accumulation in hepatocytes.

Although deficiency of the autophagy gene Atg7 or Atg5 increases hepatic fat content, there is still substantial evidence that blocking autophagic flux significantly aggravates lipid accumulation (23, 36). In this study, we demonstrated that lipid droplet accumulation induced by SRSF3 loss was due to the reduced degradation in the autophagy-lysosomal system. The blockage of autophagic flux is multifactorial including disruption of lysosome homeostasis and a fusion failure of autophagosome and lysosome (37). STX17 is a key autophagosomal SNARE protein that mediates the fusion of autophagosome and lysosome by forming STX17-SNAP29-VAMP8 complex (34). In this study, STX17 was decreased in the liver of steatosis mice and STX17 protein was reduced by the loss of SRSF3. The reduction of STX17 caused fusion failure and subsequent lipid accumulation. This observation is consistent with one report that disruption of the association of Pacer with STX17 and the homotypic fusion and protein sorting complex could abolish autophagosome maturation and lipid droplet clearance (38, 39). Few studies reported the degradation of STX17 protein. Acinetobacter baumannii upregulates LncRNA-GAS5 and promotes the degradation of STX17 by blocking the activation of YY1 and finally inhibits autophagy and aggravates inflammation (40). Whether STX17 can be degraded by the ubiquitin-proteasome system is unknown. In this study, we revealed that STX17 ubiquitination and degradation were induced by SRSF3 knockdown. We also confirmed that the upregulation of E3 ligase SIAH1 was involved in the ubiquitination and proteasomal degradation of STX17 and the accumulation of lipid droplets. SIAH1 plays a role in autophagy because it could be recruited by Synphilin-1 to the mitochondria, where it promotes mitochondrial protein ubiquitination and subsequent mitophagy (41). However, SRSF3 did not regulate the RNA stability of SIAH1. The upstream regulators of SIAH1 are complex. SIAH1 can be transcriptionally regulated by the upstream determinants (42). More research would be beneficial to clarify whether the upregulation of SIAH1 mediated by SRSF3 loss is due to transcription. In addition, the downregulated ATG5 and ATG7 proteins under SRSF3 knockdown suggest that we need to address the role of SRSF3 in autophagosome maturation in the future.

In addition to acting as a proto-oncogene or a potential suppressor in many types of cancer, the role of SRSF3 in autophagy may also be opposite in different cell models. A previous study demonstrated that SRSF3 is an autophagy suppressor in oral squamous cell carcinoma cell lines (43). Since an adapter protein p62 accumulates when autophagy is inhibited, and decreased levels can be observed when autophagy is induced, p62 may be used as an autophagy degradation marker to study autophagic flux (32). Our study found that the p62 protein increased significantly after SRSF3 knockdown in HepG2, Huh7, and THLE-3 cells. But SRSF3 knockdown reduced p62 protein in three oral squamous cell carcinoma cell lines in the study by Jia et al. (43). The inconsistent expression of p62 may be due to different cell models. The accumulation of p62 may be attributed to the nonuse of p62 under low autophagy level (scarce LC3 and accumulated p62 protein) or to the failed autophagolysosomal degradation (accumulated LC3 and p62 protein). We observed an accumulation of p62 and LC3, indicating that autophagolysosomal degradation was blocked under SRSF3 knockdown. Besides, in the study by Jia et al., SRSF3 knockdown led to an increase of both mRNA and protein of BECLIN1. However, the mRNA expression of several key genes was diverging from the protein level in our study. It suggested a complex regulatory mechanism of SRSF3 in hepatocytes. More evidence on the relationship between SRSF3 and autophagy may be needed.

SRSF3 has a certain relationship with many diseases, such as Alzheimer’s disease, glaucoma, bipolar disorder, the occurrence, and metastasis of various tumors, as well as chemotherapy resistance (44). Some SRSF3 inhibitors have shown significant anticancer efficacy, suggesting a potential therapeutic strategy for tumors. Theophylline and amiodarone downregulate SRSF3 expression and exert anticancer activity in cancer cell lines (45). The cardiotonic steroid digitoxin, long prescribed in the clinical treatment of heart failure, regulates alternative splicing through the depletion of SRSF3 (46, 47). These suggest that we should consider the side effects of these drugs on the liver for patients with these diseases.

In conclusion, we demonstrated that loss of SRSF3 in hepatocytes promoted STX17 degradation and the fusion failure of autophagosome and lysosome. In addition to the alternative splicing function, SRSF3 is an essential factor involved in liver lipophagy and beneficial for lipid droplet clearance. SRSF3 was a protective molecule in metabolic liver disease. Liver-specific overexpression of SRSF3 may be a potential therapeutic strategy for preventing NAFLD.

## Data availability

All data are available upon request to the corresponding authors (Centre for Lipid Research, the Second Affiliated Hospital, Chongqing Medical University; chenyaxi@cqmu.edu.cn, x.ruan@ucl.ac.uk).

## Supplemental data

This article contains supplemental data.

## Conflict of interest

The authors declare that they have no conflicts of interest with the contents of this article.
