# Supplementary material for: Loss of Splicing Factor SRSF3 Impairs Lipophagy Through Ubiquitination and Degradation of Syntaxin17 in Hepatocytes
Source: J Lipid Res. 2023 Feb 8;64(3):100342. doi: 10.1016/j.jlr.2023.100342 (PMC10020108; doi:10.1016/j.jlr.2023.100342)
Supplement: Supplemental Figure S1 [file mmc1.pdf]

SUPPLEMENTAL INFORMATION:

Loss of SRSF3 Impaired Lipophagy through Ubiquitination and Degradation of Syntaxin17 in Hepatocytes

Yun Li[1#], Tao Wang[1#], Qiumin Liao[1#], Xiaoting Luo[1], Xing Wang[1], Shu Zeng[1], Mengyue You[1], Yaxi Chen[1*] and Xiong Z. Ruan[1,2*]

[1]Centre for Lipid Research & Key Laboratory of Molecular Biology for Infectious Diseases (Ministry of Education), the Second Affiliated Hospital, Chongqing Medical University, 400016 Chongqing, China; [2]John Moorhead Research Laboratory, Centre for Nephrology, University College London Medical School, Royal Free Campus, University College London, London NW3 2PF, United Kingdom.

Figure S1

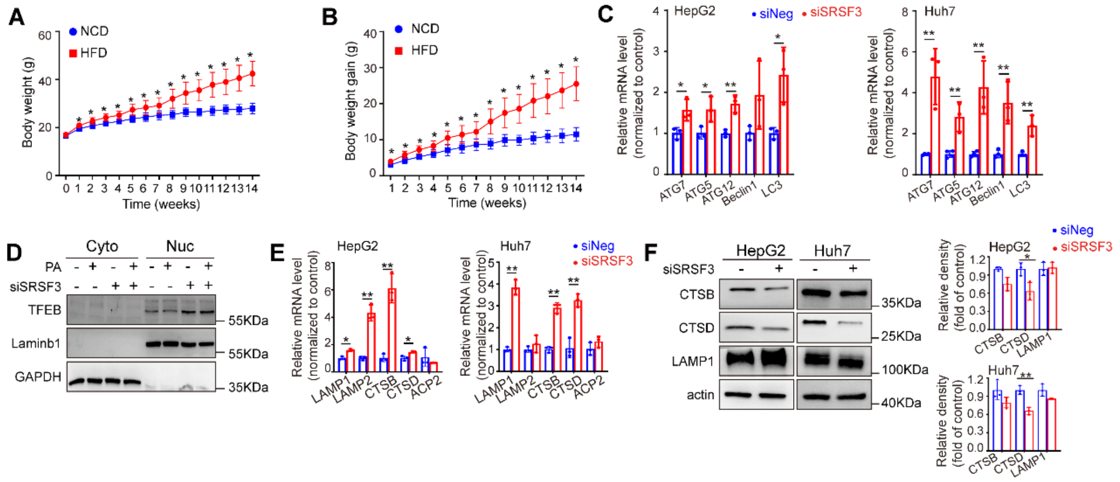

Supplemental Figure S1. Additional data. Body weight (A) and body weight gain (B) of mice fed with NCD or HFD (n=7). SRSF3 was knocked-down (n=3), and mRNA of autophagy-related genes was detected by quantitative PCR (C). (D) Analysis of TFEB in the nucleus by western blotting. mRNA (E) and protein (F) analysis of lysosome-related genes. Data are shown as individual data points and mean ± SD and * $p<0.05$, ** $p<0.01$.
