# Supplementary material for: Loss of Splicing Factor SRSF3 Impairs Lipophagy Through Ubiquitination and Degradation of Syntaxin17 in Hepatocytes
Source: J Lipid Res. 2023 Feb 8;64(3):100342. doi: 10.1016/j.jlr.2023.100342 (PMC10020108; doi:10.1016/j.jlr.2023.100342)
Supplement: Supplemental Table S1 [file mmc2.docx]

| Supplemental Table S1. List of quantitative PCR primers. | |  |
| --- | --- | --- |
| Gene | Forward | Reverse |
| homo *CTSB* | TGTGGTACCTTCCTGGGTGG | GCCACCATTACAGCCAGCAG |
| homo *CTSD* | TGCCTCTCCACTTTGACACC | CTGGACATCGCTTGCTGGAT |
| homo *LAMP1* | CAATGCGAGCTCCAAAGAAATCAA | TGTCTTGTTCACAGCGTGTCT |
| homo *LAMP2* | ACAACTCACTCCACAGGCAG | TGCAATGCTGAAAACGGAGC |
| homo *ACP2* | GGTCAGTTAACCAAGGAGGGG | GGTCAAAGTCTGTGCTTCGC |
| homo *STX17* | AAGGGGACAGGAGTCAGTCT | TGGATCGGAGTTGCTGAACTG |
| homo *SNAP29* | CCTGAACAGAATGGCACCCT | TGGGGACAGGGTCTGTATCA |
| homo *VAMP8* | GCTGCTCTGAGACATGGAGG | GCGATGTCGTCTTGAAGTGC |
| homo *SMURF1* | GCTTCAAGGCTTTGCAAGGTT | TCGATCCGGTTAAAGCAGGT |
| homo *TRIM32* | CGGGCGGTCAGGAATTTGA | CCATGCAGATGGGGCATTCT |
| homo *Cul3* | ATCCGGGCCTTTCCGATGA | TCCAGTGTAGAGCTTTTCTCCA |
| homo *UBQLN1* | TGCCTGGAGTAGGAGCTAGT | AGGATTTCCAGCAAATAGGGGA |
| homo *ELOB* | CTTCCGGGCAGATGACACC | ACCAGGCAGACTCCCAAATC |
| homo *RHOBTB2* | GGCGAGGTGGCAGTAAACAAG | GCTCACAGGGATGACAGTTGA |
| homo *RNF7* | TCCCTCAAGAAGTGGAACGC | ATCTAAGACAGGCATCCATCACC |
| homo *SIAH1* | ACTTCTCATGACGGGAAAGGC | GCAGTCTGACGGCTCATTTCTTTT |
| homo *FBXO27* | AGAACTGCTGGATAGTGGCAG | AGAACACGTGGGTGACGTGAA |
| homo *FBXO45* | GCTACAAGGCCAAGATACGTG | GCTCTGAGCAATGGGGTTTC |
| homo *SRSF3* | TGGCAACAAGACGGAATTGGA | CAAAGCCGGGTGGGTTTCTA |
| homo *ACTB* | TTCTACAATGAGCTGCGTGTG | GGGGTGTTGAAGGTCTCAAA |
| Mus *Lamp1* | CCTCTATGGCACTGCAACTG | CAGGCTAGAGCTGGCATTCATC |
| Mus *Lamp2* | CTGCCACAACCAACTTCACC | GGAAATGTTGAAAGCTGAGCCA |
| Mus *Ctsb* | AGAAGCTGTGTGGCACTGTC | CTGCCCCAAATGCCCAACAA |
| Mus *Ctsd* | CGTCCTCCTTCGCGATTATCA | CATAGTACTGGGCATCCAGG |
| Mus *Acp2* | GGTCAGCTAACCAAGGAAGGG | GGTTGGCCTCAGCACTCAT |
| Mus *Snap29* | CAGCCCAACAGCAGATTGAAA | AGAAGGTTCTTTGGGGACCG |
| Mus *Stx17* | AGGTGAAGTTACGCAGGCTT | TATTGGAGCGCAGTTGCTGA |
| Mus *Vamp8*  Mus *Srsf3* | TTGGAAGCCACGTCTGAACA  CCGGGAACTAGATGGAAGAACA | GGGATGGTACCAGTGGCAAA  ATCATCTCGAGGACGACGAC |
