# Supplementary material for: Loss of Splicing Factor SRSF3 Impairs Lipophagy Through Ubiquitination and Degradation of Syntaxin17 in Hepatocytes
Source: J Lipid Res. 2023 Feb 8;64(3):100342. doi: 10.1016/j.jlr.2023.100342 (PMC10020108; doi:10.1016/j.jlr.2023.100342)
Supplement: Supplemental Table S2 [file mmc3.docx]

| Supplemental Table S2. Primary antibodies. | | |
| --- | --- | --- |
| Name of antibody | Vendor | Cat. number |
| anti-LC3B | Sigma | L7543 |
| anti-p62 | Abcam | ab109012 |
| anti-SRSF3 | Abcam | ab198291 |
| anti-TFEB | Abcam | ab270604 |
| anti-SNAP29 | Abcam | ab181151 |
| anti-SIAH1 | Abcam | ab2237 |
| anti-LAMP1 | Abcam | ab278043 |
| anti-Rab7 | Abcam | ab137029 |
| anti-CTSD | Abcam | ab75852 |
| anti-VAMP8 | Abcam | ab76021 |
| anti-CTSB | Abcam | ab125067 |
| anti-STX17 | Gene Tex | GTX130212 |
| anti-YKT6 | Thermofisher | A305-479A |
| anti-ACC | Abcam | ab109368 |
| anti-FASN | Abcam | ab128870 |
| anti-SREBP1 | Abcam | ab28481 |
| anti-CPT1α | Proteintech | 15184-1-AP |
| anti-Beclin1 | Abcam | ab207612 |
| anti-SCD1 | Abcam | ab236868 |
| anti-ATG5 | Abcam | ab108327 |
| anti-ATG7 | Abcam | ab52472 |
| anti-ATG12 | Abcam | ab303488 |
